# Reforming Markets to Strengthen Independent Pharmacies

**DOI:** 10.1001/jamahealthforum.2025.0142

**Published:** 2025-04-04

**Authors:** Priyanka A. Abraham, Jacob T. Kannarkat, Dima Mazen Qato

**Affiliations:** Independent Scholar, Houston, Texas (Abraham); Department of Psychiatry, Yale School of Medicine, New Haven, Connecticut (Kannarkat); Program on Medicines and Public Health, Alfred E. Mann School of Pharmacy and Pharmaceutical Sciences, University of Southern California, Los Angeles (Qato); Schaeffer Center for Health Policy and Economics, University of Southern California, Los Angeles (Qato).

Independent pharmacies in rural and urban areas serving lower-income, uninsured, or publicly insured populations are significantly more likely to close than their chain pharmacy counterparts.^1^ They are also more likely than chain pharmacies to serve Black and Hispanic neighborhoods and to be the only pharmacy serving a neighborhood.^1^ Therefore, closures of independent pharmacies exacerbate the problem of “pharmacy deserts” across the country and threaten access to medications and preventive services, including vaccinations, leading to worsening nonadherence and disparities in many vulnerable communities.^1,2^

On July 9, 2024, the Federal Trade Commission released an interim report of their investigation into increasing consolidation of pharmacy benefit managers (PBMs) and insurers, describing business practices that disadvantage independent pharmacies.^3^ Although pharmacy contracts associated with low prescription reimbursements are often cited as contributing to closures, few policy efforts have addressed underlying market-level factors. In this Viewpoint, we highlight key challenges and offer policy recommendations to strengthen independent pharmacies.

## Challenges of Independent Pharmacies

### Effective Market Positioning

Relative to chain pharmacies, independent pharmacies encounter not only structural disadvantages, including greater per-unit business costs (eg, higher administrative costs from less automation) and reliance on prescription revenue leading to narrow profit margins, but also disadvantages related to effectively positioning themselves in an evolving market landscape. Consider that when the Medicare Part D program was implemented by the Centers for Medicare & Medicaid Services (CMS) in 2006, many independent pharmacies had difficulty adapting to the program’s operational requirements and lower reimbursements, contributing to closures in the years immediately following^4^ and underscoring their sensitivity to market shifts. More recently, online pharmacies with direct-to-consumer models and mail-order services represent newer competition that chain pharmacies can more effectively compete with through their own comparable offerings. Ultimately, these competitors draw patients away from the more personalized and community-based services that distinguish independent pharmacies.

Pharmacies are reimbursed for prescriptions based on contracts with PBMs, third-party administrators that manage prescription drug benefits for health plans. Because independent pharmacies are smaller than chain pharmacies and unaffiliated with vertically consolidated PBMs (ie, an insurer owning a chain pharmacy and a PBM), they have less negotiating power with PBMs. Although pharmacy services administrative organizations commonly represent groups of independent pharmacies in negotiations with PBMs and assist in evaluating business consequences of contracts, they are often unable to achieve more favorable terms.^5^ Independent pharmacies can find themselves in a double bind, wherein they must choose between entering contracts outside their financial interest or opting out and losing out on the associated patients and prescription volume.

### Preferred Network Access

The Part D program aims to provide comprehensive drug coverage to Medicare beneficiaries through Part D plans offered by sponsors (ie, PBMs or private insurers) that often establish preferred pharmacy networks. Through lower cost-sharing, these plans incentivize, or steer, patients to fill prescriptions at preferred pharmacies as opposed to in-network nonpreferred or out-of-network pharmacies. Participating in a Part D plan’s preferred network remains an important consideration for pharmacies, as Part D accounts for about one-third of national spending on prescription drugs.

Preferred networks have become prevalent outside of Part D, and evidence suggests they preferentially drive prescription volume toward PBM-affiliated pharmacies.^3^ The Federal Trade Commission report notes industry vertical consolidation as an important contributor.^3^ Independent pharmacies may be compelled to forgo participating in preferred networks due to PBM contracts offering inadequate reimbursements and unmanageable direct and indirect remuneration (DIR) fees.

### DIR Fee Management

Direct and indirect remuneration fees were introduced within the Part D program to account for payment adjustments provided to plan sponsors for drugs (eg, manufacturer rebates, pharmacy price concessions) with the intent of controlling costs. Their application, however, has expanded beyond their original purpose (eg, gaining entry to preferred networks, meeting pharmacy performance metrics) and, like preferred networks, have become widespread outside of Part D. Within Part D plans, pharmacy performance-based concessions grew to represent the second-largest category of DIR, after manufacturer rebates.^6^

Although performance metrics are reasonable to promote value-based care, their use within DIR fees has been questionable. Certain metrics are not entirely within the pharmacy’s control (eg, medication adherence), and others are misapplied (eg, generic dispensing ratio for specialty pharmacies), leading to unavoidable losses. Moreover, the ways that metrics dictate fees are often not transparent in contracts, impeding a pharmacy’s ability to prioritize quality improvement efforts. Independent pharmacies are disproportionately affected by DIR fees because they are less privy to information about DIR payment structures compared with PBM-affiliated pharmacies.^3^

Concerns over pharmacy revenue unpredictability and increased patient out-of-pocket costs recently led to a CMS rule, which in part aims to mitigate retroactively applied DIR fees in Part D plans by requiring, starting in January 2024, that they be reflected in the negotiated price the patient pays at the pharmacy (ie, at the point of sale).^6^ Although this change provides independent pharmacies some visibility into fees, it is unlikely to alleviate their financial impact.

## Policy Recommendations

### Improving Network Standards

Currently under Part D, convenient access standards require that sponsors have pharmacy networks that ensure beneficiaries can reasonably access pharmacies. These standards set thresholds for the percentage of beneficiaries living within a certain distance of an in-network pharmacy (either nonpreferred or preferred) tiered by rurality level (eg, at least 70% of beneficiaries live within 15 miles of an in-network pharmacy in rural areas). Because independent pharmacies have limited influence in negotiating contracts, requiring convenient access to preferred pharmacies specifically, as opposed to in-network pharmacies generally, may compel sponsors to offer better contract terms and promote pharmacy participation. This measure could encourage needed growth of independent pharmacies in pharmacy deserts as entrepreneurs recognize potential opportunities to contract with sponsors seeking continued compliance with requirements.

### Regulating Pharmacy Contracts

Moving DIR fees to point of sale provides greater transparency for patients and pharmacies; however, already financially challenged independent pharmacies must still confront ambiguous fees and disadvantageous arrangements with PBMs. Accordingly, CMS should mandate standardization of metrics that accurately reflect pharmacy care quality (eg, immunization rates, patient satisfaction) and enforce clarity on their use to calculate DIR fees under Part D so pharmacies can sufficiently prepare to reorganize their operations accordingly.

Even beyond Part D, generally regulating contracts between pharmacies and PBMs may help protect independent pharmacies against anticompetitive or harmful business practices. Many states have passed provisions that have withstood legal challenges. For instance, Arkansas state law requires PBMs to reimburse pharmacies at least their drug acquisition costs, preventing negative reimbursements.^7^ Consider also the pass-through model for PBMs, adopted by Medicaid programs in several states, wherein the administrative fee is the only source of revenue under the contract. This model is in opposition to the commonplace PBM practice of charging the health plan more than what the pharmacy is reimbursed for the drug (ie, spread pricing), which contributes to opaqueness of pharmacy reimbursements.^3,7^ Federal regulation mirroring successful models of state regulation should be considered to broadly improve pharmacy access to fair reimbursements and viable opportunities to participate in preferred networks.

## Conclusions

Independent pharmacies are critical in the care of vulnerable populations but are themselves vulnerable to serious financial risk and closure. Addressing the causes of their disadvantaged market position requires a thoughtful policy prescription to strengthen their ability to advocate for fair terms and keep communities in the foreground.
